# Trends and challenges in research on integrative health practices and pediatric palliative care: a bibliometric approach

**DOI:** 10.3389/fped.2025.1699130

**Published:** 2025-12-02

**Authors:** Aline Maria de Oliveira Rocha, Flávia H. Santos, João Roberto Bissoto, Claudio Arnaldo Len, Simone Brasil de Oliveira Iglesias

**Affiliations:** 1 Universidade Federal de Sao Paulo, São Paulo, Brazil; 2 Centro Universitario Sao Camilo, São Paulo, Brazil; 3 Hospital das Clinicas da Faculdade de Medicina da Universidade de Sao Paulo, São Paulo, Brazil; 4 Institute of Education, University College London, London, United Kingdom

**Keywords:** palliative care, pediatrics, child, complementary therapies, integrative medicine, bibliometrics

## Abstract

**Background:**

Approximately 21 million children and adolescents worldwide require palliative care annually. Pediatric palliative care (PPC) seeks to improve the quality of life of patients, families and caregivers through a holistic and multidisciplinary approach. The integration of palliative care with integrative medicine emphasizes the biopsychosocial well-being of patients by combining pharmacological and non-pharmacological strategies.

**Objectives:**

This study aims to explore trends and challenges in research on integrative health practices within the context of pediatric palliative care using a bibliometric approach.

**Design:**

Bibliometric analysis of the scientific literature.

**Methods:**

A bibliometric analysis was conducted using relevant databases to identify publications on integrative health practices in pediatric palliative care. Both quantitative and qualitative methods were employed to assess publication trends, key themes and research gaps.

**Results:**

Interest in the integration of palliative care and integrative medicine has grown, highlighting interventions such as therapeutic massage, aromatherapy, hypnosis, meditation/mindfulness and herbal medicine as having therapeutic potential. Nonetheless, communication challenges among patients, families and healthcare professionals remain a barrier to effective implementation.

**Conclusion:**

Integrative health practices represent promising, low-cost strategies that can enhance the quality of pediatric palliative care. Future studies should aim to develop effective implementation strategies to broaden access to these practices, thereby reducing suffering and promoting well-being in children and adolescents receiving palliative care.

## Introduction

Approximately 21 million children and adolescents require palliative care each year worldwide (1). In 2018, the International Association for Hospice and Palliative Care (IAHPC) redefined palliative care as “the active holistic care of individuals of all ages with serious health-related distress due to serious illness [..]. Its goal is to improve the quality of life of patients, families and caregivers.” (2) This definition encompasses prevention, early identification, comprehensive assessment and management of physical, psychological, spiritual and social needs, delivered with respect for individual values and beliefs, while recognizing the essential role of family (2, 3).

Pediatric palliative care (PPC) support to children throughout the entire course of a serious illness, regardless of whether curative treatment is being pursued. It requires a broad, multidisciplinary approach that engages families and leverages available community resources (3).

The early integration of palliative care has been associated with significant benefits, including improved clinical outcomes, patient well-being and overall quality of life, without precluding curative interventions (4). This underscores the critical importance of expanding access to palliative care and investing in research aimed at reducing suffering and enhancing quality of life for children and their families (3–5).

The integration of palliative care and integrative health practices has gained growing attention due to a shared emphasis on the biopsychosocial well-being of patients. For clarity and consistency, the term “integrative health practices” is used throughout this manuscript to encompass a broad range of strategies commonly referred to in the literature as *complementary*, *alternative*, or *integrative* therapies, reflecting current international trends in research and clinical application that emphase the combination of conventional and non-conventional approaches in patient-centered care.

This multidimensional approach adresses physical, social, spiritual and emotional aspects of health, combining pharmacological and non-pharmacological strategies to enhance quality of life (6–8).

In pediatrics, addressing these interconnected domains is particularly complex and requires tailored approaches. Interventions such as therapeutic massage, aromatherapy, hypnosis, meditation/mindfulness and the use of herbal remedies exemplify integrative health practices that may complement palliative care. The coordinated integration of conventional and integrative practices can be particularly valuable in situations involving substantial impairment in quality of life or functionality, especially when children and their caregivers experience distress related to the illness and/or its treatment or when symptoms prove difficult to manage (9–11).

However, effective implementation often depends on overcoming communication barriers among patients, families and healthcare providers. These practices also represent low-cost options with promising therapeutic potential for health institutions (6, 9, 10).

Despite significant advances in research on palliative care and integrative health practices individually, important knowledge gaps remain regarding their intersection and the overall landscape of scientific production in this field. Therefore, this study seeks to systematically explore the current landscape and emerging trends in the literature, a foundational overview to inform future research and guide clinical innovation in pediatric palliative care.

## Methods

This study employed a bibliometric analysis to map the scientific literature on integrative health practices in pediatric palliative care. This study adhered to the guidelines for reporting bibliometric reviews in biomedical literature to ensure transparency and comprehensiveness in the search and selection process (12).

### Information sources and search strategy

A systematic search was performed across four major multidisciplinary and biomedical databases: Scopus, Scielo (Scientific Electronic Library Online), PubMed and Embase.

The search strategy was designed to be comprehensive and was built using standardized descriptors from the Decs/MeSH vocabularies, such as“palliative care” OR “palliative medicine” AND “alternative medicine” OR “complementary therapies” OR “complementary medicine” OR “alternative therapies” AND “pediatrics” OR “child” OR “childhood”. Additionally, specific descriptors related to practice modalities were included, such as: “traditional Chinese medicine”; “naturopathy” OR “naturopathic medicine”; “anthroposophy”; “homeopathy” OR “homeopathic formularies” OR “homeopathic pharmacopoeia”; “Ayuverdic medicine”; “meditation”; “mindfulness”; “yoga”; “mind-body therapies”; “hypnosis”; “music therapy”; “phytotherapy”; “aromatherapy”.

Depending on the database, these descriptors were sometimes applied as age-related filters and the search strategy adapted to the specific syntax.

No date or language restrictions were applied. The initial search was conducted in February 2024, repeated in August 2024, and a final update was performed in January 2025 to capture the most recent publications. A manual search of the reference lists of all included studies was also performed to identify additional relevant articles.

### Eligibility criteria

Inclusion Criteria: Studies of any language and without time restrictions were considered eligible if they addressed both pediatric palliative care (or serious, life-limiting illness in children) and any form of integrative practices. Included study designs encompassed original articles observational and interventional studies, cross-sectional and longitudinal research, systematic reviews, clinical trials, cohorts studies, case series and case reports. The objective was to gather a comprehensive body of scientific evidence on the topic.

Exclusion Criteria: In addition to duplicate publications, exclusion criteria comprised dissertations, theses, conference abstracts without full text availability, editorials, letters to editor. Studies were also excluded if or integrative health practices were not applied or if the intervention was not clearly described as such. Articles focusing exclusively on populations over 18 years of age (caregivers, healthcare professionals), those unrelated to palliative care or addressing conditions not typically associated with palliative care were also excluded.

### Study selection process

All identified records were imported into the Rayyan web application Article screening was conducted using the web-based platform Rayyan (13). Afterautomated and manual duplicate removal, the titles and abstracts of all unique records were screened independently by two reviewers against the eligibility criteria.

The full texts of potentially relevant studies were then retrieved and assessed in detail for final inclusion. Any disagreements at any stage of the selection process were resolved through discussion or adjudicated by a third reviewer.

### Data standardization and analysis

Data from the included studies were extracted into a standardized Microsoft Excel spreadsheet. The extracted variables included: year of publication, authors, author affiliations and countries, journal name, article title, abstract, keywords, MeSH terms, study design, health condition focus, and the specific integrative health practices investigated.

The extracted data were imported into R statistical software (version 4.3.1) using the R Studio interface (14) for analysis. This software was used for comprehensive bibliometric mapping. The analysis included:

Descriptive Analysis**:** Calculation of publication trends over time, leading authors, countries, journals, and most frequent keywords.

Network Analysis & Science Mapping**:** Construction of co-authorship networks (countries, authors) and co-occurrence networks of keywords to identify major research themes and collaborative patterns.

Thematic Analysis**:** Keywords were standardized and then clustered to identify conceptual themes and their evolution over time.

Data visualization, standardization and the generation of graphs in comparative analyses was performed using R Studio interface. Descriptive analyses of the bibliometric indicators were presented through narrative summaries, tables, and figures, providing a comprehensive overview of the findings.

## Results: bibliometric analysis

The database searches initially identified a total of 4,801 articles. After the removal of duplicates, 3,850 unique records underwent title and abstract screening. Following this, 304 full-text articles were assessed for eligibility. A total of 284 articles met the inclusion criteria and were included in the final bibliometric analysis.

The earliest article establishing a connection between pediatric palliative care and integrative health practices was published in 1982 by Olmsted, Zeltzer and LeBaron (15) in the United States. Supported by the National Cancer Institute, this study appeared in *The Journal of Pediatrics*. The most recent publication included is from 2024 by Eran Ben-Arye et al. (16) from Israel, Germany and Norway. Published in *Current Oncology Reports* the study was supported by the Technion—Israel Institute of Technology.

**Figure 1:**
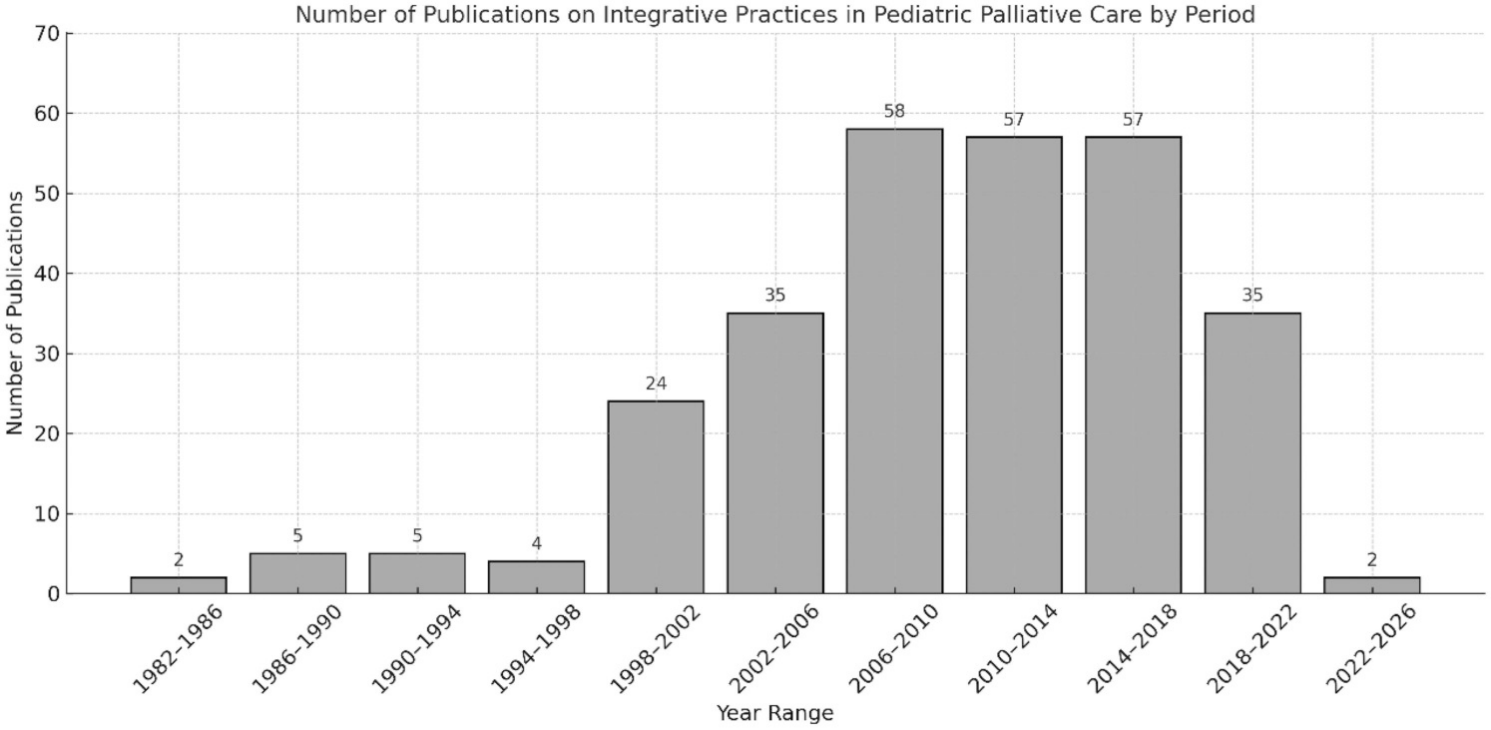
Temporal distribution of scientific publications addressing pediatric palliative care and integrative health practices, organized by five-year intervals from 1980 to 2024.

Scientific output on the topic has grown significantly over time, with 6 articles published in the 1980s, 13 in the 1990s, 101 in the 2000s, and 140 in the 2010s. The year 2018 marked the peak of annual publications, with 25 articles published. In the current decade (2020s), 26 articles have been published to date. Figure 1 illustrates the distribution of publications across each five-year interval, highlighting the temporal trends in the field.

**Figure 2:**
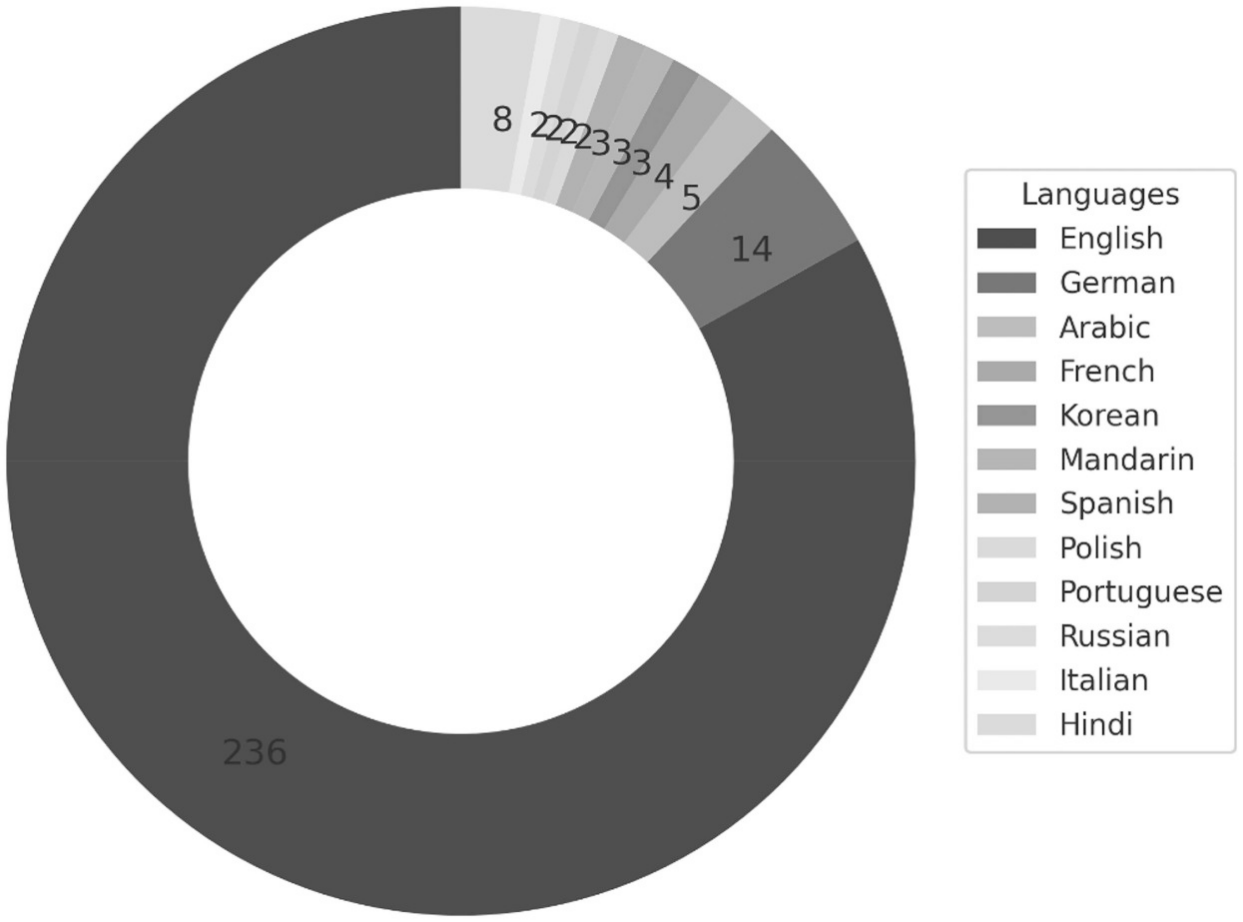
Distribution of publications by language.

English was the predominant language of publication (*n* = 245, 86.3%), underscoring its role as the lingua franca of global science. Other languages included German (*n* = 15), Portuguese (*n* = 12), French (*n* = 8), and Spanish (*n* = 4), as illustrated in Figure 2.

**Figure 3:**
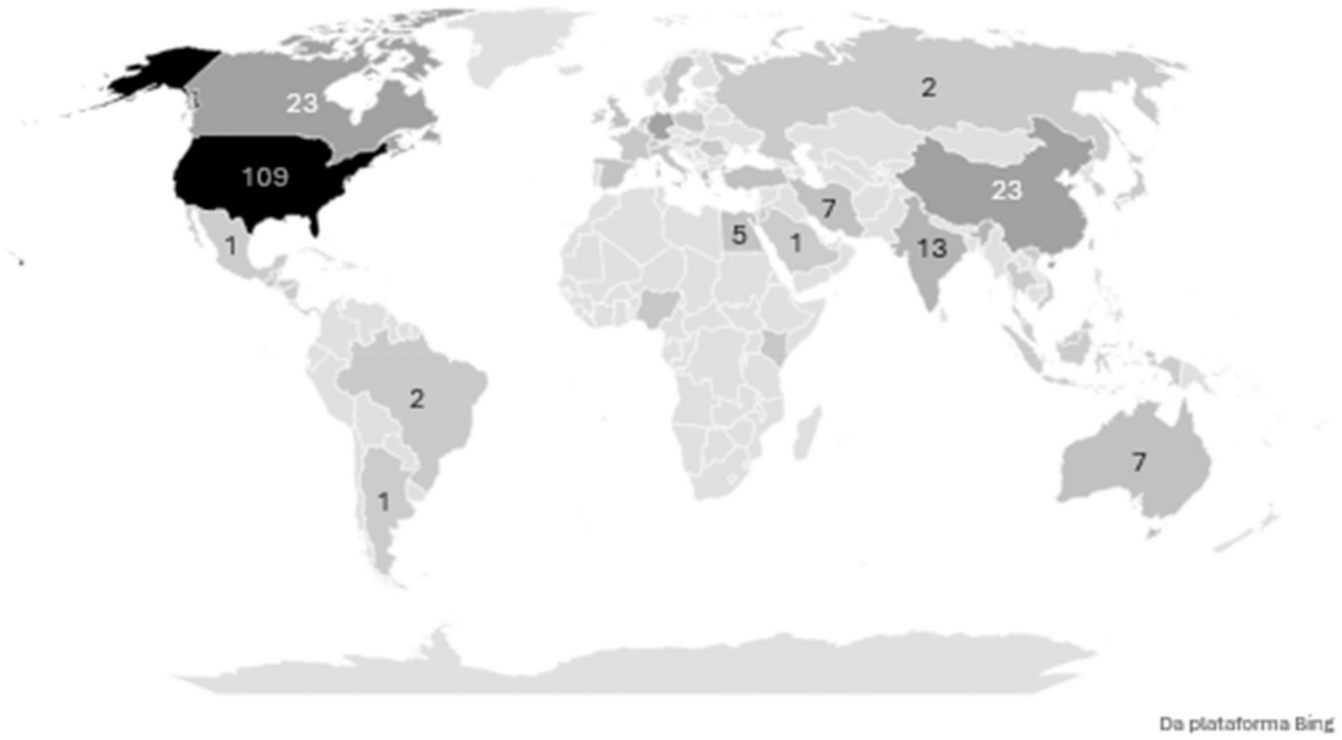
Geographic distribution of scientific publications.

The United States was the leading contributor to scientific literature on the topic, both in independent research and international collaborations, followed by Canada, Germany, the United Kingdom and Brazil, as illustrated in Figure 3.

**Figure 4:**
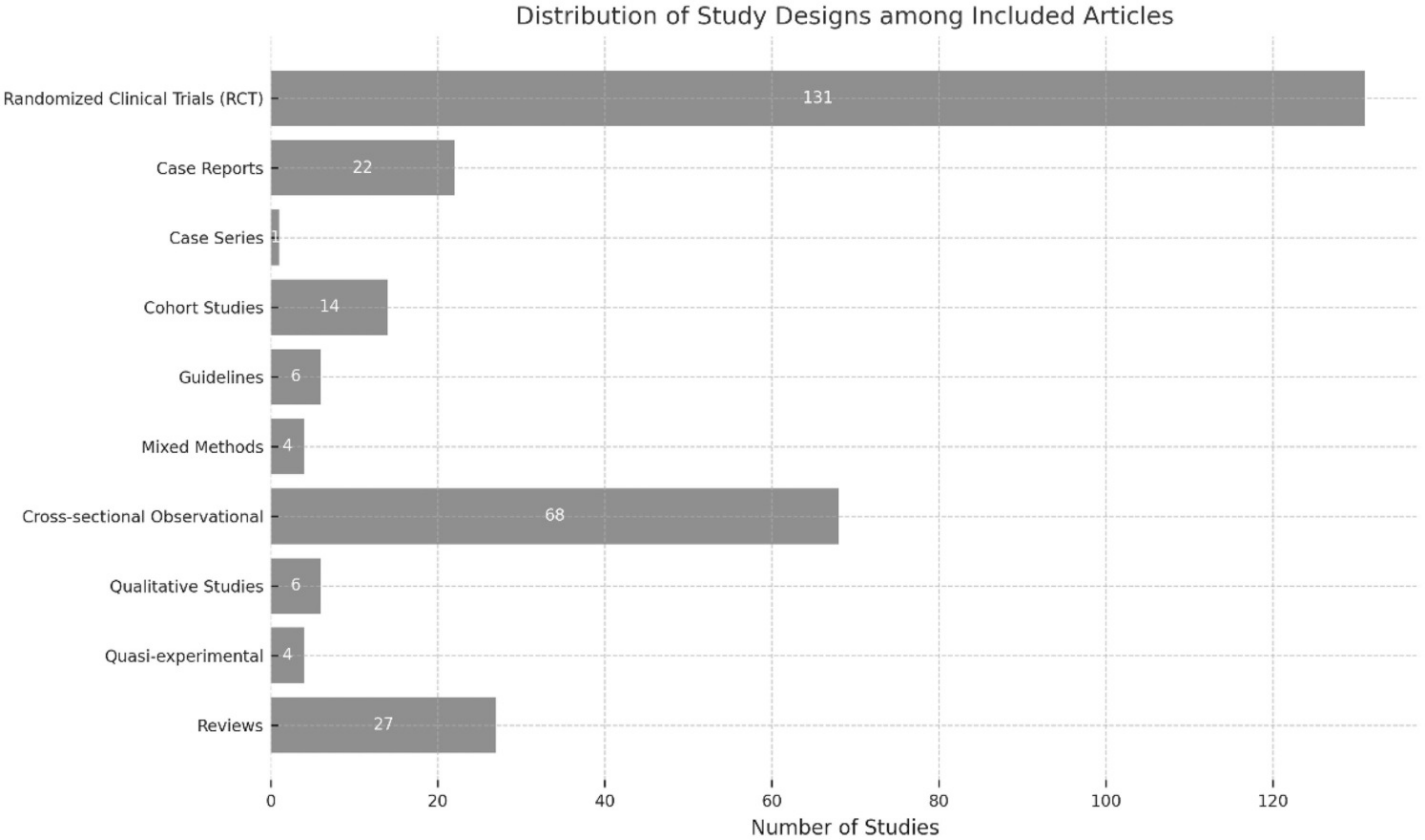
Distribution of study designs among the included publications.

A diverse range of study designs was identified. The most frequent were case reports and case series (78), followed by clinical trials (52), encompassing randomized, non-randomized, crossover and pre-post intervention designs, and observational studies (45). A significant portion of the literature also consisted of review articles (*n* = 38) and qualitative studies (*n* = 21). The distribution of study designs is detailed in Figure 4.

**Figure 5:**
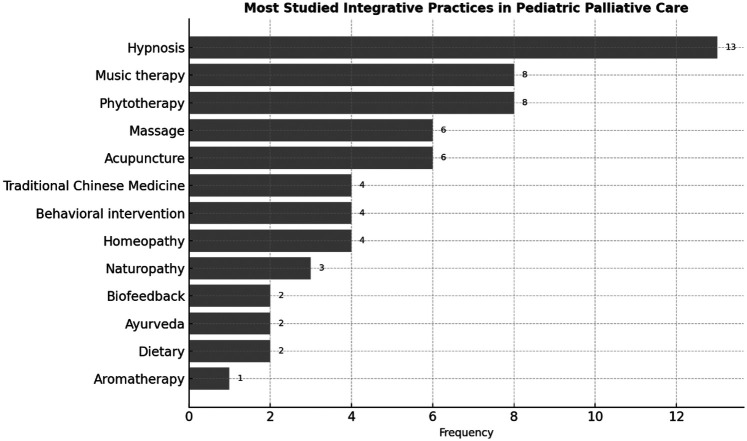
Frequency of the most studied integrative health practices addressed in the selected studies.

The most frequently investigated integrative practices were music therapy (19), therapeutic massage (19), hypnosis (14), aromatherapy (12) and herbal medicine/phytotherapy (11). Other practices were also identified, reflecting the diversity of approaches explored in the context of pediatric palliative care, as shown in Figure 5.

Analysis of the health conditions revealed a strong focus on oncological diseases (*n* = 132), followed by neurological disorders (*n* = 23) and complex chronic conditions (*n* = 21). A keyword co-occurrence analysis confirmed that “Pain” and “Pain Management” were among the most central and frequently occurring symptoms, intricately with “anxiety,” “procedures,” “hypnosis,” and “music therapy.” This highlights that symptom control, particularly pain, is a primary research driver in this field.

The primary journals publishing on this topic were predominantly within the field of pediatric onco-hematology, with notable contributions from *Journal of Pediatric Oncology Nursing*, *Journal of Pediatric Hematology/Oncology* and *Pediatric Blood & Cancer*. These were followed by general pediatric journals, including *The Journal of Pediatrics*, *Children's Health Care*, *Paediatrics* and *Acta Paediatrica*, as well as oncology-focused journals such as *Journal of Clinical Oncology*, *Psycho-Oncology* and *Cancer*. Additionally, specialized journals in integrative health practices, such as the *American Journal of Clinical Hypnosis*, *The International Journal of Clinical and Experimental Hypnosis* and *Journal of Music Therapy*, also made significant contributions.

The most frequently used MeSH terms and keywords were “palliative care,” “pediatrics,” “complementary therapies,” “pain management” and “quality of life.” However, 71 articles did not include associated MeSH terms or keywords. Most article keywords were provided in English, French, German or Portuguese. The overall distribution of MeSH terms aligned with the pattern described above.

Some additional keywords from articles published in other languages were identified, expanding the thematic scope of the review:

- French: Douleur, Enfant, Adolescent, Traitements non medicalmenteux, Cancer; food supplements; Child; Homéopathie; Interaction; alternative and complementary methods; phytotherapie.
- Spanish: Masaje; Toque terapéutico; Estimulación táctil; Recién nacidos prematuro.
- German: Komplementäre und alternative Behandlungsmethoden, Homöopathie, Akupunktur, Anthroposophische Medizin, Phytotherapie
- Portuguese: Dor; massagem terapêutica; criança; câncer

**Figure 6:**
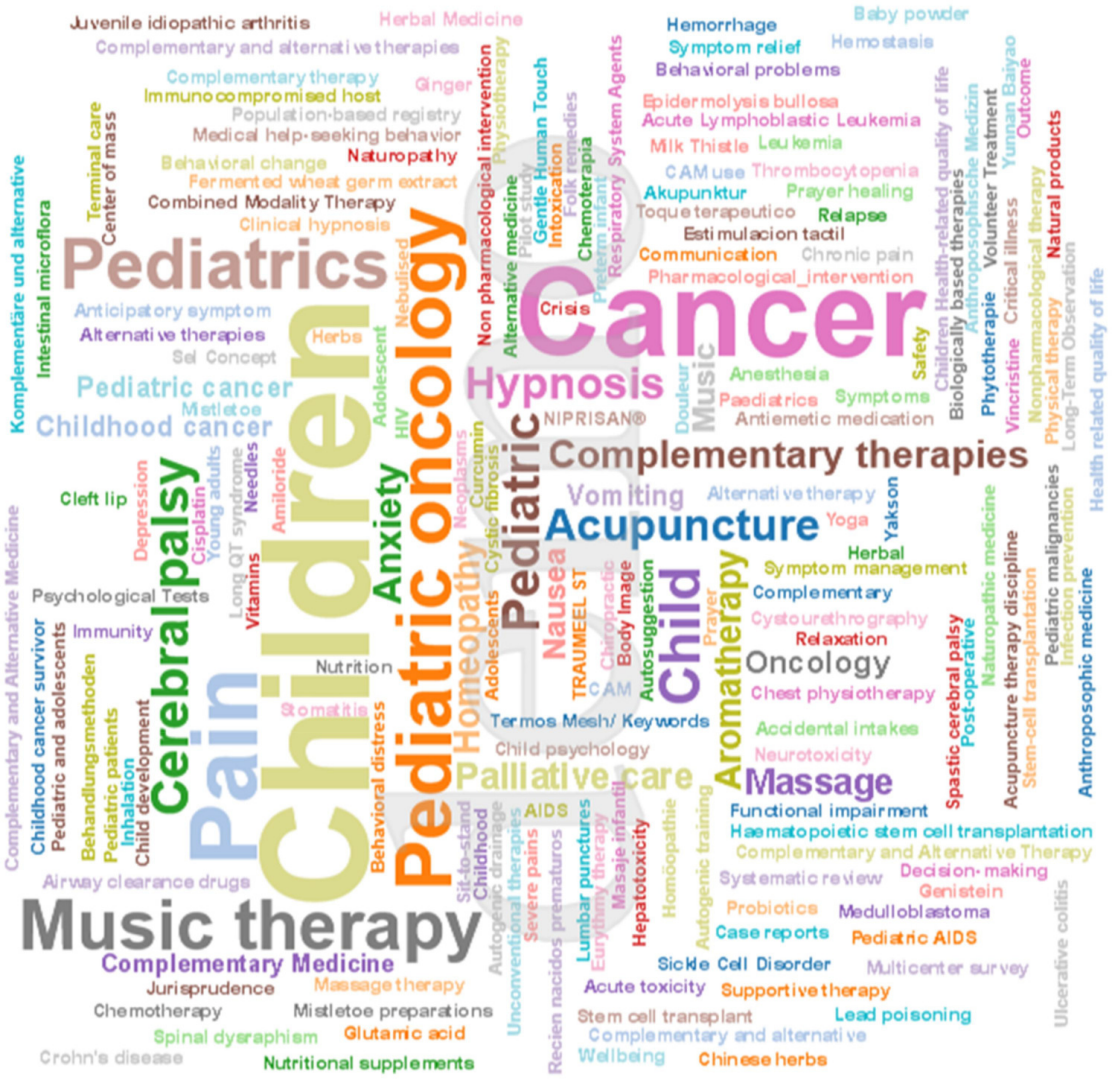
Word cloud of MeSH terms and keywords from the multilingual included studies. The size of the word corresponds to its frequency.

A word cloud generated from the keywords of all 284 studies visually emphasizes the dominance of these terms (Figure 6). Multilingual keywords (“Douleur” in French, “Dor” in Portuguese, “Schmerz” in German) further reinforced the global focus on pain and symptom management.

The most prolific and cited author in this field was L. Zeltzer, best known for the highly influential article “*Hypnosis and nonhypnotic techniques for reduction of pain and anxiety during painful procedures in children and adolescents with cancer,”* which has received 341 citations to date. According to a 2025 search on the Scopus platform, Zeltzer holds an H-index of 79, with 407 published documents and a total of 21,716 citations. A network analysis of co-authorship revealed she maintains extensive collaboratations with other leading researchers, including J. Tsao (117 joint publications), L. Robison (65 co-authored articles), S. Evans (48 co-authored articles), Laura C. Seidman (38), A. Mertens (33) and S. LeBaron (32), indicating a strong, established research community in this niche.

## Discussion

This bibliometric analysis provides a comprehensive mapping of the scientific literature concerning in pediatric palliative care and integrative health practices. By analyzing 284 publications over four decades, our study elucidates the evolution, key areas of focus and significant gaps in this emerging field. The findings not only chart the intellectual landscape but also offer valuable insights for future research, clinical practice and policy development (Tables 1, 2) (17, 18).

**Table 1:** Comprehensive search strategy by database.

Database	Date of last search	Search string	Records retrieved
PubMed	January 15, 2025	[“palliative care"[MeSH] OR “palliative medicine"[MeSH]] AND [“complementary therapies"(MeSH) OR “integrative medicine” OR..] AND [“pediatrics"(MeSH) OR “child” OR..]	1,850
Scopus	January 15, 2025	[TITLE-ABS-KEY (“palliative care”)] AND [TITLE-ABS-KEY (“complementary therapy” OR “music therapy"..)] AND [TITLE-ABS-KEY (pediatr* OR child*)]	1,723
Embase	January 15, 2025	'palliative therapy'/exp AND ‘complementary medicine'/exp AND ‘child'/exp	1,105
Scielo	January 15, 2025	[tw:(“cuidados paliativos”)] AND [tw:(“terapias complementares” OR “medicina integrativa”)]	123
Total			**4,801**

**Table 2:** Categorization of studies by thematic area.

Related to health condition					
Neurological	Oncological	Complex chronic diseases	Age range	Disease phase	Type of Care/Other
Cerebral palsy (16), spinal dysraphism (1), muscular dystrophy (2), epilepsy (3), neuromuscular diseases (1)	Neoplasms (52), including pediatric cancer (9), pediatric oncology (20), childhood cancer survivors (10), oncology (34), acute lymphoblastic leukemia (5), neuroblastoma (1), lymphoma (1), retinoblastoma (1)	Cystic fibrosis (4), pediatric HIV/ AIDS/ (6), sickle cell disease/ crisis (2), Down syndrome (1), epidermolysis bullosa (4), type 1 diabetes mellitus (1), congenital heart disease (1), complex chronic conditions (1), parental HIV infection (1), Crohn's disease (2), ulcerative colitis (1), inflammatory bowel disease (3), juvenile idiopathic arthritis (3), connective tissue diseases (1)	Pediatrics (74); child development/ psychology/ infants (71), adolescence (16), young adults (3), preterm (2), children's health (1)	Inpatient, terminal care (1), health care utilization (1), disease severity (1), functional impairment (1), rehabilitation (3), palliative care (13), hospice (2), critical illness (1), chronic illness (1), comprehensive function (1), nursing (3), death (1), pediatric intensive care unit (1)	Pediatric illnesses (1), hematology (3), developmental disabilities (1), health care/ health care workers/ utilization (3), Progressive and nonprogressive diseases (1)
Related to pharmacological treatments					
Niprisan, Traumeel, methotrexate	Medication (4), Drug utilization (1)	Pharmacological intervention (1)	Airway clearance agents (expectorants, mucolytics) (1)	Antiemetic medications (2)	Amiloride (1)
Related to comparative non-pharmacological treatments					
Harmful **vs.** non-harmful therapies (1), physician communication (1)	Inhalation (1), nebulization (1), communication (4), nonpharmacological therapy (2), intervention (1)	Psychological experiences (1), psychological behaviors (5)	Health-related quality of life (8), exercise (2), EMLA (1), physical therapy (4)	Chest physiotherapy (1), respiratory therapy (1), user computer interface (1), video games (1)	Medical help-seeking behavior (1) behavioral distress/changes (4),
Related to integrative practices					
General descriptors	Body-mind practices	Biology/diet based therapies	Complete medical systems	Manipulative/body-based	Others
Alternative therapy (9), complementary and alternative medicine/ CAM/ complementa treatments (53), complementary medicine (16), complementary therapies (13), unconventional therapies (5), alternative therapies (6), supportive care (7), holistic medicine (1), biologically based therapies (1), oral CAMs	Music therapy (19), homeopathy (11), hypnosis (14), mind–body approaches (1), relaxation (3), eurythmy therapy (1), mindfulness/meditation (7)	Honey (2), black seed oil (1), mistletoe (3), herbal medicine (11), dietary supplements (1), nutrition (3), folk remedies (1), vitamins (1), supplements (3), nutritional supplements (1), organic acids (1), ph (1), Milk Thistle (1), phytotherapy (2), fermented wheat germ extract (1)	Naturopathy (2), anthroposophic (5), prayer healing (1), homeopathic (2), traditional Chinese medicine (4), ayurveda (5), Glutamic acid (1), polyphenols (1), terpenes (1), Yunnan Baiyao (1), ginger (1), curcumin (1), hyperbaric oxygen (1), osteopathy (2), udwartana (1), traditional medicine (1)	Yakson (1), chiropractic (3), yoga (5), massage (19), chiropractic (3), qi qong (1), natural health products (1), Propolis (1), Agnimandya (1), personal health practices (1), touch (1), Samvardhana ghrita (1), medicinal plants (1)	Acupuncture (1), probiotics (1), Intestinal microflora (1), food therapy (1), Acacia Senegal (1), healing Touch (2), biofield therapy (1), acoustic stimulation (1), Phakka roga (1), prayer (3), natural products (1), aromatherapy (12), Panchkarma procedures
Related to symptoms					
Gastrointestinal/dermatological	**Psychological/cognitive**	**Hematological/infectious**	**General symptomatology**	Pain	Other
Stomatitis (1), mucositis (2), fatigue (4), peristomal dermatitis (2), stoma care (1), nausea (10), vomiting (9)	Autosuggestion (1) anxiety (11), quality of life (9), psychological tests (1), anticipatory symptom (1), depression (3), voiding (1), body image (1), sleep (5), distress (4), decision-making (1)	Febrile neutropenia (2), thrombocytopenia (1), infection prevention (1) hemorrhage (1), hemostasis (1)	Self Concept (1), symptom relief (1), psychosocial issues (1), symptoms (2), wellbeing (1), symptom management (5)	Chronic pain (3), Pain Management (8), acute/ postoperative pain (36), pediatric pain (1)	Immunity (1), Muscle tone (1), motor disabilities (1), constipation (1), immunocompromised status (1)

Related to interventions or adverse events					
Procedures	Toxicity/safety	Anesthesia/vaccination	Stress/surgery	Cardiac events	Medications
Lumbar puncture (1), bone marrow transplantation (1), haematopoietic/ stem cell transplantation (8)	Neurotoxicity (1), quality of life (1), lead poisoning (1), autogenic training (1), hepatotoxicity (2), acute toxicity (1), safety (2)	Anesthesia (2), complications (1), vaccination (1), post-operative care (1)	Cystourethrography (1), medical procedures (1), stress (11), craniofacial surgery (1), venipuncture (1)	Torsades de Pointes (1), accidental ingestion(1), arrhythmias (1), Long QT syndrome (1)	Vincristine (1), chemotherapy (7), cortisol (2), intoxication (1), cisplatin (1), side effects (1)
Relating to the type of study and methodologies					
Methodologies	CAM research	Pilot and registries	Records and reports	Surveys/trials	Other
Qualitative methods (1), practice guidelines (1)	CAM-related research (2), systematic reviews (2), qualitative studies (1), outcomes (2)	Pilot studies (3), population-based registry (1)	Cleft lip (1), medical records (1), case reports (1), multicenter survey (1)	Cross-sectional studies (1), survey design (1), randomized controlled trial (5),	Written self-disclosure (1)
Miscellaneous/other topics					
General	Social/economic	Rare conditions	Psychological/functional	Movement/performance	Additional
Relapse (1), twinning programs (1), feasibility (2), treatment-related experiences (1)	Policy (1), Mucopolysaccharidosis (1), socioeconomic experiences (1), resistance training (1), socioeconomic factors (1), grounded theory (1)	Substrate reduction therapy (1), genistein (1), jurisprudence (1), disabled children (1), meridians (1)	Behavioral issues(8), sit-to-stand (1), inherited conditions (1), post-traumatic stress (2), resilience (1)	Center of mass (1), patterned sensory enhancement (1), immigrant parents (1)	Interactions (1), actigraphy (1), trends (1), GMFM (1), mood (1), health psychology (1)

It is important to contextualize our findings within the methodological framework of bibliometric analysis, that quantifies and maps the literature itself. Our results, therefore, reflect research activity and scientific discourse rather than the strength of clinical evidence. The predominance of case reports and small-scale trials indicates a field still in its relative infancy, building its foundational knowledge through anecdotal evidence and pilot studies before progressing to large-scale randomized controlled trials. The rigorous and transparent methodology employed, adhering to specific guidelines, ensures that this map is a reliable representation of the current scientific conversation.

Despite the growing integration of integrative health practices in the context of pediatric palliative care research on this subject remain scarce. This aligns with global shifts toward patient-centered care that addresses biopsychosocial and spiritual distress (2, 6).

The pronounced focus on pediatric oncology, as opposed to other life-limiting conditions, likely reflects the higher availability of research funding and infrastructure within oncology, as well as the high symptom burden associated with intensive cancer treatments (19–22).

Furthermore, the most frequently studied practices—such as therapeutic massage, hypnosis, music therapy and aromatherapy—share key characteristics: they are generally non-invasive, low-risk and easier to integrate alongside conventional treatments with minimal conflict and into existing palliative care protocols. Their prominence in the literature suggests they are perceived as more readily acceptable and implementable within mainstream clinical settings.

These disparities reveal important gaps in the evidence base that may skew clinical adoption and guideline development. Therefore, expanding research on less-studied but widely used practices is essential to ensure a more equitable and evidence-informed approach to integrative pediatric palliative care.

The implementation of integrative health practices in pediatric palliative care has been most frequently documented in studies originating from countries such as the United States, Canada and several European nations. This pattern likely greater institutional acceptance and systematic incorporation of such approaches into healthcare delivery systems (6, 23, 24). However, while it might be expected that regions where traditional and integrative health practices are culturally embedded would produce higher volume of research output, this trend was not consistently observed in the present analysis (25, 26).

The striking concentration of publications from high-income countries unveils a critical gap and reflects a combination of structural and systemic factors. This disparity is likely multifactorial, stemming from inequities in research funding, institutional capacity and barriers to publishing in high-impact, English-language journals (10, 27).

Additionally, the regulatory environment in these countries may facilitate ethical approval and institutional support for studies involving integrative health practices. Cultural acceptance of such therapies within clinical settings may also influence both the implementation and academic interest in documenting these approaches. This is a crucial finding, as it suggests that the global evidence base does not fully represent the rich traditions of integrative and traditional medicine prevalent in many low- and middle-income countries. Future efforts must prioritize funding and initiatives that support research capacity in low- and middle-income countries, where the burden of life-limiting pediatric conditions is often highest and the cultural acceptance of high-income countries is deeply rooted (26, 27).

The majority of identified studies focus on the use of integrative health practices in children with neurological disorders, oncological conditions or complex chronic illnesses. Within pediatric oncology, these approaches have gained increasing acceptance, likely due to the recognized adverse effects of conventional treatments, which substantially affect patient's quality of life (25–28).

Among the most frequently employed interventions are dietary modifications, vitamin supplements and herbal therapies, which are generally considered simple and more easily accepted by children and their caregivers.

While the primary aim of this study was to map research trends, some patterns can offer preliminary guidance for clinical decision-making. The most frequently studied integrative health practices, such as music therapy, hypnosis, and aromatherapy, are not only prominent in the literature but also appear across multiple study types and patient populations.

These interventions are generally non-invasive, low-cost and adaptable to pediatric palliative care settings, which may explain their popularity. Although definitive clinical recommendations cannot be made based solely on bibliometric frequency, the concentration of studies in these areas suggests they are more likely to be supported by emerging evidence and clinical feasibility. Thus, pediatric palliative care teams seeking to integrate such practices may consider starting with these modalities while advocating for further research to strengthen the evidence base.

To date, systematic reviews and randomized controlled trials have identified significant limitations in the integration of integrative health practices into pediatric palliative care, largely due to methodological challenges. The inherently individualized and holistic nature of these interventions complicates their evaluation through conventional clinical trials designs, while the development of adequate placebos for therapies such as acupuncture, massage or aromatherapy remains a persistent obstacle. Furthermore, the lack of strict regulation and standardization of herbal remedies and nutritional supplements contributes to variability in both dosage and quality, which can affect study outcomes and limit reproducibility (26–28).

Based on our mapping, future research should move beyond descriptive and pilot studies. Priority areas include conducting well-designed RCTs for the most promising practices to establish efficacy; investigating the best strategies to integrate these evidence-based into standard PPC protocols, addressing training, workflow, and cost-effectiveness; focus on Underrepresented Populations, directing research attention beyond oncology to include children with neurological, metabolic and genetic disorders requiring palliative care; and actively researching the barriers and facilitators to integrative practices use in low- and middle-income countries and underserved communities.

Addressing these barriers is essential for advancing the understanding and responsible integration of integrative health practices within pediatric palliative care.

## Limitations

This study has several limitations. First, as a bibliometric analysis, it reports on the volume and relationships within the literature but does not assess the methodological quality of risk of bias of the individual studies included. As a result, the present findings reflect patterns in research activity and interest rather than the strength or reliability of the evidence itself.

Second, while our search was comprehensive across major databases, it is possible that relevant studies in non-indexed journals or books were missed.

Third, the inherent English-language and publication bias in major databases may have led to the underrepresentation of relevant research published in other languages, despite our inclusion of multilingual keywords. This limitation is especially relevant in light of our findings that regions with strong cultural traditions in integrative health practices are often underrepresented in the international literature.

Fourth, although citation counts were used to assess research visibility, this analysis did not explore potential citation bias, self-citation practices or temporal citation effects. These elements should be considered in future bibliometric studies for a more nuanced understanding of scientific impact.

## Conclusion

This bibliometric analysis offers a seminal map of the scientific landscape at the intersection of integrative health practices and pediatric palliative care. By systematically quantifying and analyzing four decades of research, this study moves beyond a simple narrative review to provide an objective, data-driven overview of the field's evolution, core themes, and collaborative networks.

Our findings confirm a steadily growing global interest in this holistic approach, predominantly led by research institutions in high-income countries. The field is characterized by a focus on non-invasive, readily implementable modalities such as music therapy, hypnosis and therapeutic massage, primarily in the context of pediatric palliative care and pain management.

However, this map also reveals critical uncharted territories. The significant geographical disparities in research production, the predominance of preliminary study designs and the underrepresentation of complex traditional systems highlight a field with immense potential for growth. The concentration of evidence for certain practices provides a foundational platform for clinical innovation, while the identified gaps serve as a strategic guide for future scientific inquiry.

To advance the field, stakeholders must now leverage this map. Clinicians can use it to identify promising practices for pilot implementation in their settings. Researchers are directed toward the urgent need for robust trials, implementation science, and studies in underrepresented populations and disease groups. Finally, policymakers and funders are provided with a clear justification for investing in global research equity and capacity building in this area. Ultimately, this bibliometric synthesis not only documents the past and present of the field but also provides a crucial compass for its future, guiding efforts to reduce suffering and enhance the quality of life for children living with life-limiting conditions and their families.

## Data Availability

The datasets presented in this study can be found in online repositories. The names of the repository/repositories and accession number(s) can be found in the article/supplementary material.
